# Comparison of the Prediction of Effective Moment of Inertia of FRP Rebar-Reinforced Concrete by an Optimization Algorithm

**DOI:** 10.3390/ma16020621

**Published:** 2023-01-09

**Authors:** Nag-Seop Jang, Young-Hwan Kim, Hong-Seob Oh

**Affiliations:** Department of Civil Engineering, Gyeongsang National University, Jinju 52725, Republic of Korea

**Keywords:** FRP rebars, effective moment of inertia, deflection, optimization algorithm, harmony search algorithms

## Abstract

FRP (fiber-reinforced polymer)-reinforced concrete members have larger deflection than reinforced concrete members because of the low modulus of elasticity of the FRP bar. In this paper, we proposed a new effective moment of inertia equation to predict the deflection of FRP-reinforced concrete members based on the harmony search algorithm. The harmony search algorithm is used to optimize a function that minimizes the error between the deflection value of the experimental result and the deflection value expected from the specimen’s specifications. In the experimental part, four GFRP (Glass Fiber-Reinforced Polymer)- and BFRP (Basalt Fiber-Reinforced Polymer)-reinforced concrete slab specimens were manufactured and tested. FRP-reinforced concrete slabs were reinforced with GFRP and BFRP rebars on spiral rib surfaces. The effects of the FRP reinforcement ratio and balanced reinforcement ratio ($\rho_{f}$/$\rho_{fb}$), the moment of inertia of the transformed cracked section and the gross moment of inertia ($I_{cr}$/$I_{g}$), and the cracking moment and the maximum service load moment ($M_{cr}$/$M_{a}$) on the effective moment of inertia have been considered. The experimental results and predicted results of the flexural testing of concrete slabs reinforced with FRP rebars were compared, and the experimental results were in good agreement with the calculated values using the proposed effective moment of inertia equation.

## 1. Introduction

Reinforced concrete structures are economical, can freely manufacture the shape and size of members, and are efficient in terms of maintenance. Therefore, concrete and steel rebar are recognized as the most essential materials in the construction industry [1]. As reinforced concrete structures are exposed to various environments, the steel rebar corrodes when moisture seeps into the concrete [2]. The corrosion of steel rebars in reinforced concrete structures can seriously affect the safety and durability of structures in harsh environments [3,4]. Therefore, the use of FRP (Fiber-Reinforced Polymer) rebar can be an effective solution to secure the performance and increase the service life of concrete structures [5]. Research is being actively conducted on the development and application of various types of FRP rebars, such as GFRPs (Glass Fiber-Reinforced Polymers), BFRPs (Basalt Fiber-Reinforced Polymers), AFRPs (Aramid Fiber-Reinforced Polymers), and CFRPs (Carbon Fiber-Reinforced Polymers) [6].

FRPs have excellent advantages such as high tensile strength, non-corrosiveness, and light weight compared with steel rebars. When FRPs are used as a steel rebar substitute, it is possible to prevent the deterioration of concrete structures caused by the corrosion of steel and increase their durability [7,8,9]. Therefore, FRPs are being used more and more in various civil structures such as bridges, tunnels, highways, marine structures, and underground structures [10].

FRP rebars do not have a yield point, but rather exhibit complete elastic behavior until failure. In addition, FRP rebars have a relatively low modulus of elasticity compared to steel rebar [11,12,13]. FRP rebars bond to concrete differently than steel rebars because their surface geometries and mechanical properties are different from steel rebars [14]. Therefore, FRP-reinforced concrete members have larger deflections and crack widths than reinforced concrete members with the same reinforcement ratio because of the difference between the physical and mechanical properties of FRP rebars [15,16,17].

In addition, FRP rebar-reinforced concrete members have brittle failure modes in flexure, either due to concrete crushing or sudden ruptures in the FRP rebar [18]. Concrete crushing failure is preferred since it allows for control over deflection and cracking and prevents the sudden rupture of the FRP rebar [19,20]. Therefore, the design of FRP-reinforced concrete members generally uses the serviceability limit state considering deflection and crack width rather than the ultimate limit state [21,22,23]. As a result, a method of predicting the expected load deflection of FRP-reinforced members with significant accuracy is required.

In this study, a modified effective moment of inertia equation was proposed, and a comparison was performed with the flexural test results of FRP-reinforced concrete slabs. The proposed effective moment of inertia was developed based on Branson’s equation. The harmony search algorithm was used, where 135 data points were used to minimize the test deflection value when the proposed equation reached the final strength. In order to examine the validity of the effective moment of inertia equation proposed through the harmony search algorithm, a comparative analysis was performed with the flexural stiffness results using concrete slabs reinforced by GFRP and BFRP. In the remainder of this paper, first, the previous model of the effective moment of inertia proposed by other researchers was analyzed. Next, the effective moment of inertia was proposed using data collected from other from other literature. FRP-reinforced concrete specimens with GFRP and BFRP rebar were manufactured and compared with the experimental results in order to verify the proposed effective area moment of inertia.

## 2. Effective Moment of Inertia and Predictive Proposal Model

### 2.1. Effectivemonet Moment of Inertia

**Figure 1:**
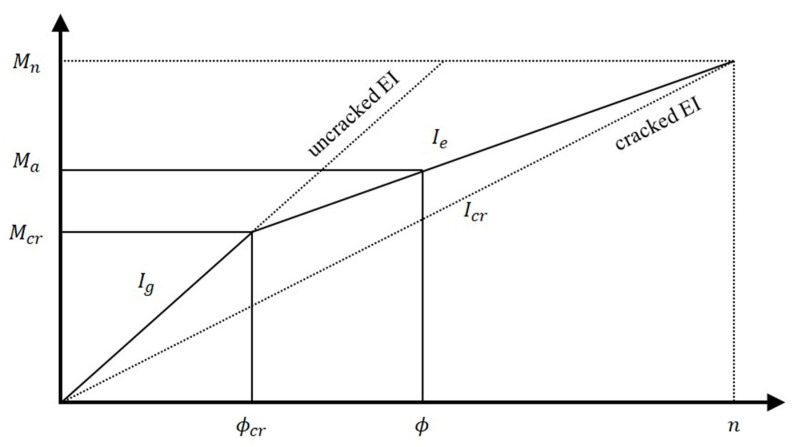
Bilinear moment–curvature relationship of an FRP-reinforced concrete section.

The relationship between the flexural moment and a curvature is defined as shown in Equation (1), wherein the flexural stiffness of a member changes depending on the magnitude of the force acting on the member, the modulus of elasticity actually changes depending on the stress level, and the moment of inertia also changes depending on the presence or absence of cracks. Figure 1 shows the effect on the size and load of the section, etc., and expresses it as an idealized moment–curvature relationship. If the load is small, the maximum moment generated will be small, and the tensile stress in the ultimate tensile section will be less than the modulus of the failure of concrete. In this case, the entire cross-section determines the stiffness of the concrete member [24]:

(1) $$\Phi =\frac{M}{\mathrm{EI}}$$

When the service load or a greater load is put into action, a flexural tensile crack is formed in the center of the member, and the position of the neutral axis in the cracked section is shifted to the compression side. At that time, only the cracked transformed section, excluding the concrete crack surface, becomes valid for determining the stiffness of the member, and the moment of inertia of the central section of the member is changed to the moment of inertia of the cracked transformed section. However, since the moment of inertia outside the central section where the flexural crack does not occur and the moment of inertia of the section with a low stress impact is assumed to be the same as the area moment of inertia, the effective moment of inertia is located between the moment of inertia of the cracked section and the area moment of inertia. According to ACI 318 [25], the effective moment of inertia after a crack occurs, proposed by Branson [26], is presented as show in:

(2) $$I_{e}={\left(\frac{M_{cr}}{M_{a}}\right)}^{3}I_{g}+\left(1-{\left(\frac{M_{cr}}{M_{a}}\right)}^{3}\right)I_{cr}\leq I_{g}$$

where $M_{cr}$ is the cracking moment, $M_{a}$ is the maximum service load moment, $I_{cr}$ is the moment of inertia of the transformed cracked section, and $I_{g}$ is the gross moment of inertia:

(3) $$M_{cr}=\frac{0.62\lambda \sqrt{f_{c}^{\prime }}I_{g}}{y_{t}}$$

(4) $$I_{cr}=\frac{bd^{3}}{3}k^{3}+n_{f}A_{f}d^{2}{\left(1-k\right)}^{2}$$

(5) $$I_{g}=\frac{bh^{3}}{12}$$

where *λ* is a modification factor reflecting the reduced mechanical properties of concrete, $f_{c}^{\prime }$ is the specified compressive strength of concrete, $y_{t}$ is the distance from the centroidal axis of the gross section, k is the ratio of the depth of the neutral axis to the reinforcement depth, $n_{f}$ is the ratio of the modulus of elasticity of FRP rebars to the modulus of elasticity of concrete, and $A_{f}$ is the area of FRP reinforcement.

(6) $$n_{f}=\frac{E_{f}}{E_{c}}$$

(7) $$k=\sqrt{2\rho_{f}n_{f}+{\left(\rho_{f}n_{f}\right)}^{2}}-\rho_{f}n_{f}$$

(8) $$\rho_{f}=\frac{A_{f}}{bd}$$

Here, $E_{f}$ is the modulus of elasticity of FRP rebar, $E_{c}$ is the modulus of elasticity of the concrete, and $\rho_{f}$ is the FRP reinforcement ratio.

Branson’s equation overestimates the stiffness of the members when the $I_{g}/I_{cr}$ of the concrete members is 3 or 4. In general, an FRP-reinforced concrete member has an $I_{g}/I_{cr}$ between 5 and 25, which can overestimate the tensile strength and underestimate the deflection and was thus found to be unsuitable for FRP-reinforced concrete members [27,28]. Therefore, as the deflection of FRP-reinforced concrete members has been shown to differ from the deflection of existing reinforced concrete members, various researchers have proposed new predictive models.

In the study of Benmokrane et al. [28], the composite action between concrete and FRP rebar may not be as perfect as is commonly assumed. Therefore, a flexural test of the FRP-reinforced concrete member reinforced with GFRP rebar was conducted using the reinforcement ratio as a variable. According to the test results, the following effective moment of inertia equation applied with the parameters $I_{g}$ and $I_{cr}$ was proposed:

(9) $$I_{e}={\left(\frac{M_{cr}}{M_{a}}\right)}^{3}\frac{I_{g}}{7}+0.84\left(1-{\left(\frac{M_{cr}}{M_{a}}\right)}^{3}\right)I_{cr}\leq I_{g}$$

Toutanji and Saafi [29] found that the order of the effective moment of inertia depends on the low modulus of elasticity of the FRP as well as the FRP reinforcement ratio. Therefore, the following equation was proposed:

(10) $$I_{e}={\left(\frac{M_{cr}}{M_{a}}\right)}^{m}I_{g}+\left(1-{\left(\frac{M_{cr}}{M_{a}}\right)}^{3}\right)I_{cr}\leq I_{g}$$

where $m=6-10\rho_{f}E_{f}/E_{s}$ for members reinforced with GFRP when $\rho_{f}E_{f}/E_{s}<0.3$.

Hall and Ghali [30] and the ISIS Canada Design Manual [31] proposed similar effective moments of inertia based on the concept of the moment–curvature relationships in the CEP-FIP model code and the assumption that the tension stiffening factor relates to the ratio $M_{cr}/M_{a}$. The proposed equation of Hall and Ghali [30] is shown in Equation (11), and the proposed equation of the ISIS Canada Design Manual [31] is shown in Equation (12):

(11) $$I_{m}=\frac{I_{g}I_{cr}}{\left(I_{g}+\beta_{1}\beta_{2}{\left(\frac{M_{cr}}{M_{a}}\right)}^{2}\left(I_{cr}-I_{g}\right)\right)}\leq I_{g}$$

(12) $$I_{e}=\frac{I_{g}I_{cr}}{\left(I_{cr}+\left(1-0.5{\left(\frac{M_{cr}}{M_{a}}\right)}^{2}\right)\left(I_{g}-I_{cr}\right)\right)}\leq I_{g}$$

where $\beta_{1}$ is a coefficient characterizing the bonding properties of rebar and is equal to 1.0 for a ribbed bar and 0.5 for a smooth bar, and $\beta_{2}$ is a coefficient characterizing the type of loading and is equal to 0.8 for the initial loading and 0.5 for sustained or cyclic loading.

ACI 440.1R-03 [32], Yost et al. [33], and ACI 440.1R-06 [34] suggest models of effective moment of inertia that are generally the same. The parameter $\beta_{d}$ accounts for the bond properties and modulus of elasticity of the FRP rebar:

(13) $$I_{e}={\left(\frac{M_{cr}}{M_{a}}\right)}^{3}\beta_{d}I_{g}+\left(1-{\left(\frac{M_{cr}}{M_{a}}\right)}^{3}\right)I_{cr}\leq I_{g}$$

ACI 440.1R-03 [32] set $\beta_{d}=\alpha_{b}\left(E_{f}/E_{s}+1\right)$, where $\alpha_{b}$ is a bond-dependent coefficient; $\alpha_{b}$ has been found to be 0.5 for GFRP rebars. Based on test results from 48 GFRP-reinforced concrete beam specimens tested by Yost et al. [33], the prediction of the model was observed to overestimate the test results. They then suggested a modification parameter $\alpha_{b}=0.064\left(\rho_{f}/\rho_{fb}\right)+0.13$. Based on an evaluation of the test results from several studies, ACI 440.1R-06 [34] proposed a new expression for $\beta_{d}=0.2\left(\rho_{f}/\rho_{fb}\right)$, where $\beta_{d}$ is mainly dependent on the relative FRP reinforcement ratio.

Rafi and Nadjai [35] compared the theoretical deflection of concrete beams reinforced with FRP rebar with the test results. Based on one set of test results, they suggested an effective moment of inertia in which the parameter $\beta_{d}$ is similar to the expression used by the ACI 440.1R-06 [34]:

(14) $$I_{e}={\left(\frac{M_{cr}}{M_{a}}\right)}^{3}\beta_{d}I_{g}+\frac{I_{cr}}{\gamma }\left(1-{\left(\frac{M_{cr}}{M_{a}}\right)}^{3}\right)\leq I_{g}$$

The parameter γ is a relationship obtained via a linear regression analysis of the test results, where $\gamma =\left(0.0017\rho_{f}/\rho_{fb}+0.8541\right)\left(1+E_{f}/2E_{s}\right)$.

Bischoff [36,37] proposed the concept of tension-stiffening in the existing Branson’s approach to present a new expression of the effective moment of inertia that can be equally applied to steel rebar and FRP-reinforced concrete beams. This model captures the bending behavior of FRP-reinforced concrete beams and develops the effective moment of inertia, which is a weighted average of the flexibility of uncracked and cracked concrete:

(15) $$I_{e}=\frac{I_{cr}}{1-\left(1-\frac{I_{cr}}{I_{g}}\right){\left(\frac{M_{cr}}{M_{a}}\right)}^{2}}\leq I_{g}\;$$

Bischoff and Gross [38] modified the previously suggested effective moment of inertia. They concluded that a reduced cracking moment equal to 80% of the cracking moment value in the ACI 318-08 [25] code provides a reasonable estimate of deflection for FRP-reinforced concrete beams using their expression [22]:

(16) $$I_{e}=\frac{I_{cr}}{1-\gamma \left(1-\frac{I_{cr}}{I_{g}}\right){\left(\frac{M_{cr}}{M_{a}}\right)}^{2}}\leq I_{g}$$

(17) $$\gamma =\frac{3\left(\frac{L_{a}}{L}\right)-4\left(4\left(\frac{M_{cr}}{M_{a}}\right)-3\right){\left(\frac{L_{a}}{L}\right)}^{3}}{3\left(\frac{L_{a}}{L}\right)-4{\left(\frac{L_{a}}{L}\right)}^{3}}\leq I_{g}$$

In Equation (17), γ is a parameter in four-point flexural beams.

Mousavi and Esfahani [22] used the genetic algorithm to propose an effective moment of inertia of GFRP-reinforced concrete beams. Their proposed effective moment of inertia presented accurate estimates, especially at high reinforcement ratios:

(18) $$I_{e}=0.17{\left(\frac{M_{cr}}{M_{a}}\right)}^{m}I_{g}+0.94\left(1-{\left(\frac{M_{cr}}{M_{a}}\right)}^{m}\right)I_{cr}\leq I_{g}$$

Neuyen et al. [39] proposed an equation of the effective moment of inertia using an AI technique called gene expression programming (GEP). They concluded that the proposed models provide good predictions of deflections of FRP-reinforced beams in comparison with experimental data and results from several existing design codes:

(19) $$I_{e}=0.80\left(0.80\frac{I_{cr}}{I_{g}}+0.14\left(\frac{I_{cr}}{I_{g}}+\frac{M_{cr}}{M_{a}}\right)\left(0.29\frac{I_{cr}}{I_{g}}\frac{\rho_{f}}{\rho_{fb}}+\frac{I_{cr}}{I_{g}}+\frac{M_{cr}}{M_{a}}\right)\right)\leq I_{g}$$

### 2.2. Proposal of Effective Moment of Inertia

In this study, FRP-reinforced flexural test data obtained by various researchers using four-point loading methods was collected to evaluate the accuracy of the effective moment of inertia equations presented in the literature and to present a new equation of the effective moment of inertia. The collected data comprised a wide range of test data, including 135 data points, and these data points were obtained from the load–deflection relationships of approximately 112 FRP-reinforced concrete members. Details of the various experimental studies are summarized in the Table 1 and Appendix A. In these data points, a wide range of changes in the modulus of elasticity of concrete FRP rebar, the compressive strength of concrete, the tensile strength of FRP rebar, the relative reinforcement ratio, the level of loading, and the ratio of the moment of inertia of the transformed cracked section to the gross moment of inertia are present. The changes in these parameters are presented in Table 2. FRP-reinforced concrete members that are out of range of the data points may not have adequately predicted deflection.

The midspan deflection of the concrete member of the four-point loading method can be calculated as shown in Equation (20), and the effective moment of inertia is the main factor in determining the deflection along with modulus of elasticity:

(20) $$\delta_{max}=\frac{PL_{a}}{48E_{c}I_{e}}\left(3L^{2}-4L_{a}^{2}\right)$$

where P is the applied load, L is the span of the beam, and $L_{a}$ is the distance between the support and the load point. Using the deflection of the member and the corresponding load, the experimental value of the effective moment of inertia may be expressed as shown in Equation (21):

(21) $${\left(I_{e}\right)}_{exp}=\frac{P_{exp}L_{a}}{48E_{c}\delta_{exp}}\left(3L^{2}-4L_{a}^{2}\right)$$

where $P_{exp}$ is the experimental load and $\delta_{exp}$ is the experimental midspan deflection corresponding to $P_{exp}$. When the experimental value of the effective moment of inertia is expressed using Branson’s Equation (2), the expression can be reversed to derive the expression for the parameter m:

(22) $${\left(I\right)}_{exp}={\left(\frac{M_{cr}}{M_{a}}\right)}^{m}I_{g}+\left(1-{\left(\frac{M_{cr}}{M_{a}}\right)}^{m}\right)I_{cr}\leq I_{g}$$

(23) $$m=\frac{Log\left(\frac{{\left(I_{e}\right)}_{exp}-I_{cr}}{I_{g}-I_{cr}}\right)}{Log\left(\frac{M_{cr}}{M_{a}}\right)}$$

**Figure 2:**
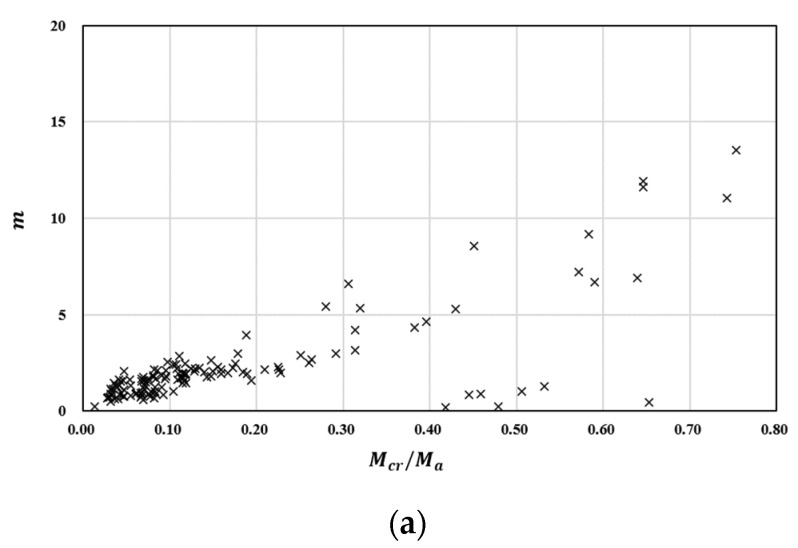

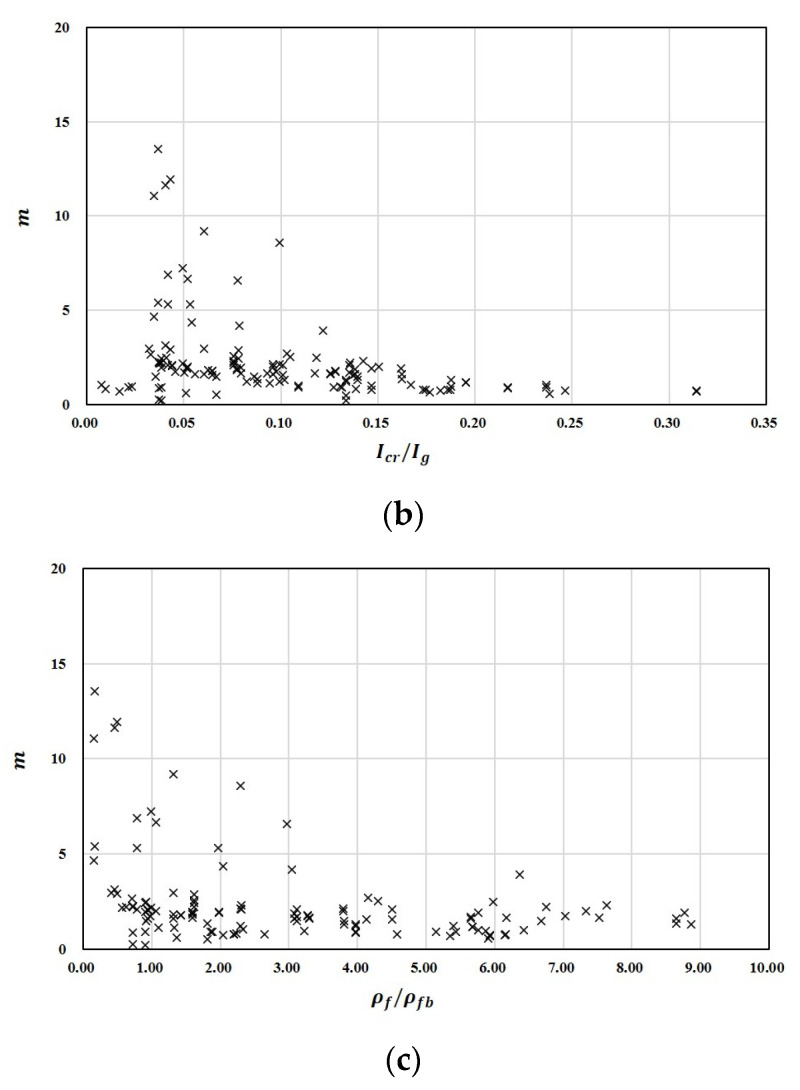
Relationship of m versus influence factor: (**a**) $M_{cr}/M_{a}$; (**b**) $I_{cr}/I_{g}$; (**c**) $\rho_{f}/\rho_{fb}$.

To derive the value of parameter m, the correlation between the $M_{cr}/M_{a}$, $\rho_{f}/\rho_{fb}$, and $I_{cr}/I_{g}$ relationships are presented in Figure 2a–c. As shown in Figure 2a, the lower the ratio of $M_{cr}/M_{a}$, the lower the value of m. In addition, as shown in Figure 2b,c, the parameter m is relatively dependent on $\rho_{f}/\rho_{fb}$ and $I_{cr}/I_{g}$. According to Branson’s Equation (2), when the load increases, the moment of inertia is interpolated between the area moment of inertia and the moment of inertia of the cracked transformed section. Thus, in Branson’s Equation (2), the reduction factor must be multiplied to estimate an effective moment of inertia value that is smaller than the moment of inertia of the cracked transformed section.

The harmony search algorithm applied in this study is the most optimized algorithm that mimics musical harmony. This is the process by which each tone harmonizes to create an optimal chord. The harmony search algorithm is characterized by the fact that it does not require mathematical differentiation processes as in other algorithms and that it is optimized by approaching it from a probabilistic perspective. The harmony value generated in the initial full set range is stored in harmony memory, and the ranking is continuously improved to derive the optimal harmony value. In this process, the HMCR (Harmony Memory Considering Rate), which is the probability of randomly generating new chords, provides the possibility of finding a better optimal value without falling into the local solution (i.e., the local optimum). In addition, the PAR (Pitch Adjusting Rate) improves the performance of the HMS (Harmony Memory Size) by considering it as a value adjacent to the existing solution in order to find a good solution [54].

In this paper, Mousavi and Esfahani’s [22] approach, which is based on Branson’s Equation (2), was followed. The proposed effective moment of inertia equation was derived using the harmony search algorithm equipped with the parameters of the experimental data of other researchers and the experimental results achieved in the present study. MATLAB R2021b has been used to generate the code for the harmony search algorithm. The objective function was to minimize the error between the deflection value of the flexural test result and the expected deflection value applying the proposed effective moment of inertia. Parameter m considers the FRP reinforcement ratio and balanced reinforcement ratio ($\rho_{f}/\rho_{fb})$, the moment of inertia of the transformed cracked section and the gross moment of inertia ($I_{cr}/I_{g})$, and the cracking moment and the maximum service load moment ($M_{cr}/M_{a})$:

(24) $$function\;equation:e=\left|\frac{\delta_{exp}-\delta_{proposal}}{\delta_{proposal}}\right|\times 100$$

(25) $$\delta_{proposal}=\frac{PL_{a}}{48E_{c}{\left(I_{e}\right)}_{proposal}}\left(3L^{2}-4L_{a}^{2}\right)$$

(26) $${\left(I_{e}\right)}_{proposal}=X_{1}{\left(\frac{M_{cr}}{M_{a}}\right)}^{m}I_{g}+X_{2}\left(1-{\left(\frac{M_{cr}}{M_{a}}\right)}^{m}\right)I_{cr}\leq I_{g}$$

(27) $$m_{proposal}=X_{3}+X_{4}\frac{\rho_{f}}{\rho_{fb}}+X_{5}\frac{I_{cr}}{I_{g}}+X_{6}\frac{M_{cr}}{M_{a}}$$

The harmony search algorithm continues until the error converges to the lowest point. For optimization, the size of HMS was set to 50, the number of interactions was set to 100,000, and the HMCR and PAR values were set to 0.70 and 0.25, respectively. The values of obtained by the harmony search algorithm through this optimization are as follows:

(28) $${\left(I_{e}\right)}_{proposal}=0.12{\left(\frac{M_{cr}}{M_{a}}\right)}^{m}I_{g}+0.77\left(1-{\left(\frac{M_{cr}}{M_{a}}\right)}^{m}\right)I_{cr}\leq I_{g}$$

(29) $$m_{proposal}=0.87-0.19\frac{\rho_{f}}{\rho_{fb}}+8.67\frac{I_{cr}}{I_{g}}+1.56\frac{M_{cr}}{M_{a}}$$

## 3. Experimental Program

**Figure 3:**
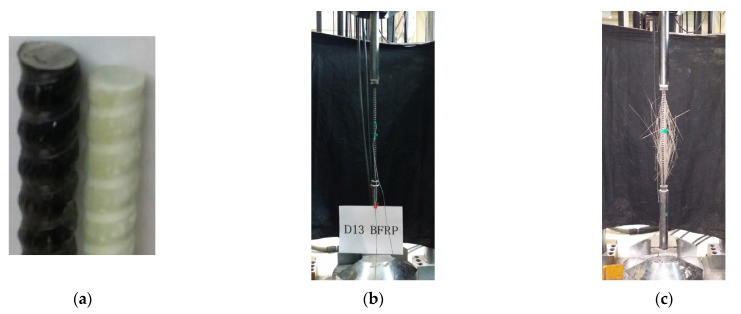
FRP rebar and tensile test: (**a**) Surface of GFRP and BFRP; (**b**) Test set up for tensile test; (**c**) Failure mode of FRP rebar.

In this study, the FRP rebar used consists of individual fibers and epoxy resins and has a spiral ribbed surface type. The FRP rebar’s diameter was 13 mm. Figure 3 shows the surface of the GFRP and BFRP rebars and the tensile test view, and Table 3 provides the properties of the GFRP and BFRP rebars. The tensile properties of the FRP reinforcement were determined by testing five GFRP and BFRP specimens according to the ASTM D 7205 standard. The tensile tests were carried out using an actuator with a capacity of 3000 kN at a rate of 3 mm per minute until the rebar failed in tension. The guaranteed tensile strengths of the GFRP rebar and BFRP rebar with standard deviation were calculated to be 839.1 MPa and 755.5 MPa, respectively. The designed tensile strength was calculated by multiplying the environmental reduction factor (0.7, for external exposure) in compliance with ACI 440.1R-15 [5], resulting in a tensile strength of 587.4 MPa and 528.9 MPa for GFRP rebar and BFRP rebar, respectively. Their moduli of elasticity were found to be 49.0 GPa and 50.5 GPa, within the general range of the modulus of elasticity for GFRP and BFRP rebar. The standard designed compressive strength of concrete applied in the experiment was 45.0 MPa, and the compressive strengths of five concrete specimens were measured, and the average compressive strength was 45.4 MPa.

**Figure 4:**
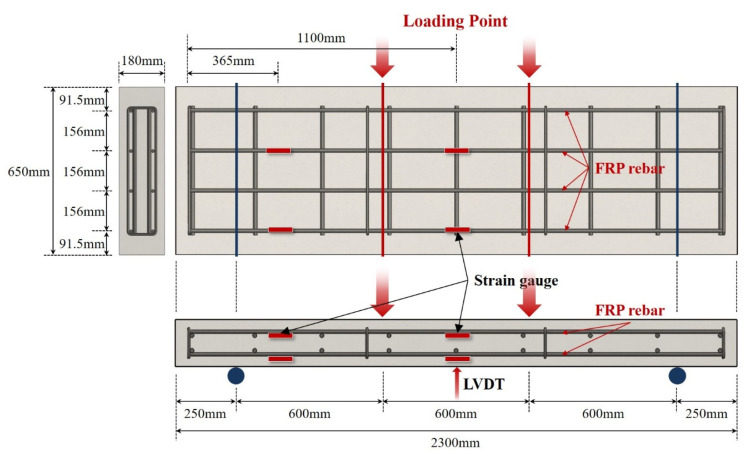
FRP-reinforced concrete slab section.

The FRP-reinforced concrete member was designed as a one-way slab in which the FRP rebar was laid transversely. The deflection was analyzed according to the effective moment of inertia in terms of the FRP rebar type and the FRP reinforcement ratio. As shown in Figure 4, the specimen has the width and height of 650 × 180 mm, a cover of 46.5 mm, a total length of 2300 mm, and a pure span of 1800 mm (the blue circle in the Figure 4). The flexural test was performed by placing a reaction force hinge at a distance of 250 mm from both ends in a four-point loading method. The actuator device was used to apply the load at a rate of 2 mm per minute at a distance of 300 mm from the center of the upper part of the specimen to both sides (the red line in the Figure 4). Data of the load and deflection were measured to determine the behavior of the one-way slab in response to the applied load. The applied load was measured through a load cell attached to the actuator, and the experimental midspan deflection value was measured using an LVDT (Linear Variable Displacement Transducer). The load data and deflection data of each FRP-reinforced member were automatically collected by a TDS-303 data logger device. Data of the load and deflection were measured once per second.

Table 4 shows the balanced reinforcement ratio design moment of each FRP reinforcement in the design section suggested by ACI 440.1R [5]. In the case of short-term behaviors, such as static experiments, it is judged that it is appropriate to analyze the strength to evaluate the behavior without considering the environmental reduction factor.

## 4. Comparison of Test Results

**Figure 5:**
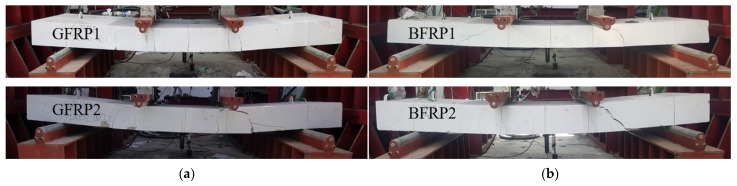
Failure state of FRP-reinforced concrete specimens: (**a**) GFRP specimen; (**b**) BFRP specimen.

Figure 5 visually represents the failure mode of GFRP and BFRP specimens after the flexural test. Table 5 shows the experimentally and analytically obtained flexural moment, crack spacing, and mode of failure of the GFRP and BFRP specimens. The FRP-reinforced specimens exhibited linear behavior before the cracking load, and after the initial cracking, it behaved linearly with the load fluctuations. After that time, brittle behavior occurred at the time of failure. The load fluctuation that occurs in the flexural test is determined to be caused by the partial rupture of the fibers in the rebar and the bonding of the concrete after the initial cracking load. The crack spacings of the GFRP and BFRP specimens were observed to be 150 to 250 mm and 150 to 200 mm, respectively.

**Figure 6:**
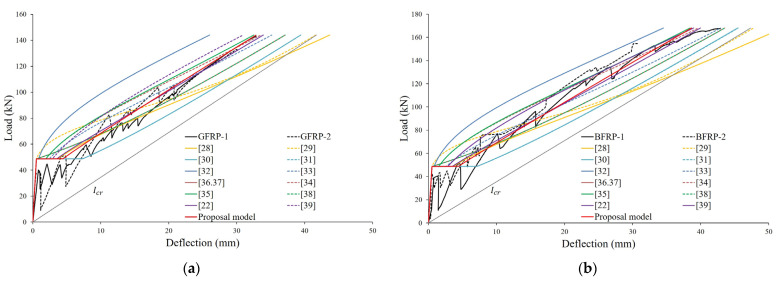
Comparison of analytical load–midspan deflection of FRP-reinforced concrete specimens: (**a**) GFRP-reinforced concrete specimens; (**b**) BFRP-reinforced concrete specimens.

Figure 6a,b compare the experimental midspan deflections for GFRP and BFRP specimens to the deflections predicted using different proposed models of the effective moment of inertia. In this study, the service load is assumed to be 40% of the ultimate load. Figure 6a shows that for the GFRP specimen corresponding to the balanced reinforcement ratio, the models of effective moment of inertia proposed by ACI 440.1R-03 [32], ACI 440.1R-06 [34], and Nguyent et al. [39] were found to underestimate the values of all states of the load after cracking. The model of Toutanji and Saafi [29] was shown to underestimate deflection at the service load stage by predicting too much stiffness after the initial load but overestimating the ultimate load. Figure 6b shows that for BFRP specimens corresponding to the compression-controlled section, only the model of ACI 440.1R-03 [32] was found to underestimate the values in all states of the load after cracking. Regardless of the FRP reinforcement ratio of the specimens, the models of Hall and Ghali [30] and the ISIS Canada Design Manual [31] showed the most conservative deflections. The model of Benmokrane et al. [28] predicts similar deflection for all specimens at the initial load but tends to overestimate the deflection the most at the ultimate load.

**Figure 7:**
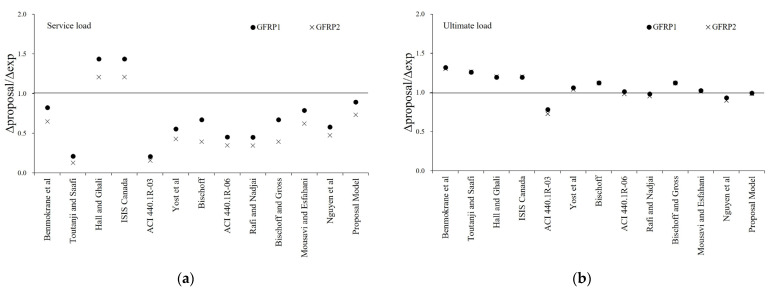
Experimental versus predicted midspan deflection of GFRP Specimens: (**a**) Ratio of experimental and predicted deflection at service load; (**b**) Ratio of experimental and predicted deflection at ultimate load.

**Figure 8:**
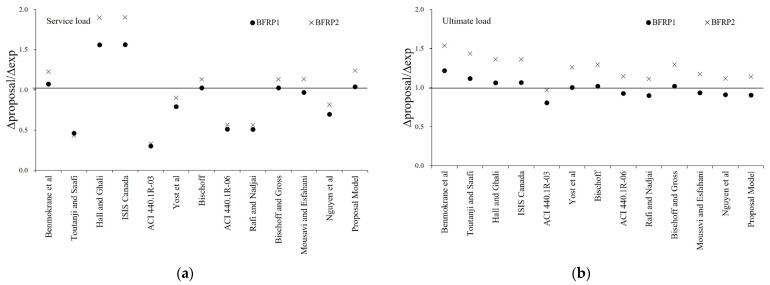
Experimental versus predicted midspan deflection of BFRP Specimens: (**a**) Ratio of experimental and predicted deflection at service load; (**b**) Ratio of experimental and predicted deflection at ultimate load.

Figure 7 and Figure 8 show the ratio of experimental and predicted deflection under service load and the ultimate load of GFRP and BFRP specimens. In the service load state, it can be confirmed that ratio of experimental and predicted deflection by the proposed effective moment of inertia is more accurate than that of the existing model. In the ultimate load state, it was found that the ratio of experimental and predicted deflection was minimized for each specimen, and it has been shown to predict the correct deflection.

Table 6 and Table 7 show the service loads of the GFRP and BFRP specimens compared with the calculation of the experimental deflection and predicted deflection for the maximum load. For GFRP specimens, the equations of Hall and Ghali [30] and ISIS Canada [31] overestimate the same value at the service load state, whereas our proposed model predicts the most accurate evaluation. In the ultimate load state, most proposed models, including the ones by Benmokrane et al. [28], overestimate the deflection. The models of ACI 440.1-06 [34] and Mousavi and Esfahani [22], as well as ours, predicted a rather accurate error with an average deflection within 1 mm. For BFRP specimens, the equations of Bishoff [36,37], Bischoff and Gross [38], Mousavi and Esfahani [22], and our proposed model predicted a rather accurate error, with the average deflection within 1 mm. In the ultimate load state, models from ACI 440.1R-06 [34], Rafi and Nadjai [35], and Nguyen et al. [39] accurately evaluated the average deflection error within 1 mm. It is judged that the fluctuation is large because the deflection generated under the same load is different for each GFRP and BFRP specimen.

## 5. Conclusions

In this paper, various proposed models for the effective moment of inertia of FRP-reinforced concrete members were reviewed. Data were secured by listing 12 existing proposal equations of the effective moment of inertia and collecting literature reporting results of the four-point flexural test. The collected data used a wide range of test data, totalling 135 data points, and these data points were obtained from the load–deflection relationship of approximately 112 FRP-reinforced concrete members. Based on the collected data and experimental results, a new equation of effective moment of inertia was proposed using the harmony search algorithm. The proposed equations of the effective moment of inertia were derived to minimize the difference between the deflection of the experimental results and the calculated value. The effects of the FRP reinforcement ratio and the balanced reinforcement ratio ($\rho_{f}$/$\rho_{fb}$), the moment of inertia of the transformed cracked section and the gross moment of inertia ($I_{cr}$/$I_{g}$), and the cracking moment and the maximum service load moment ($M_{cr}$/$M_{a}$) were considered as the parameters applied to the proposed effective moment of inertia equation.

- The proposed model considering the ratio of the reinforcement ratio and the balanced reinforcement ratio, the ratio of the moment of inertia of the transformed cracked section and the gross moment of inertia, and the ratio of cracking moment and the maximum service load moment were confirmed to have a higher accuracy than previous models.
- In the case of GFRP specimens, Mousavi and Esfahani [22] and the proposed model were the most accurate deflection at the ultimate load stage, and in the case of BFRP specimens, Neuyen et al. [39] and the proposed model were the most accurate deflection at the ultimate load state.
- The proposed model using the harmony search algorithm showed a low error in the deflection of FRP-reinforced concrete slabs. The accuracy of the proposed model was verified by experimental results and showed good agreement.
- It is necessary to verify the suitability of the proposed model for calculating the effective moment of inertia of FRP-reinforced concrete members, such as in the presence of various surface geometries, mechanical properties, and types of FRP rebar.

## Figures and Tables

**Table 1:** Experimental studies of reinforced concrete members.

Reference	Number of Specimens	Number of Data Points
Barris et al. [21]	12	12
Aiello and Ombres [40]	3	3
Erfan et al. [41]	6	12
Minkwan Ju et al. [42]	3	3
Mousavi and Esfahani [22]	9	9
Rafi et al. [43]	2	4
Toutanji and Deng [44]	6	6
El Rafai et al. [45]	3	9
Alsayed et al. [46]	4	8
Khorasani et al. [47]	16	16
Yoon et al. [48]	2	2
Nawy and neuwerth [23]	12	12
Saleh et al. [49]	9	12
Theriault and Benmokrane [50]	5	5
Thamrin et al. [51]	2	2
Goldston et al. [52]	6	6
Abdelkarim et al. [53]	8	8
Current study	4	8
Total	112	135

**Table 2:** Range of parameter changes in experimental data points.

Parameter	Minimum	Maximum
Compressive strength of concrete (MPa)	47.3	97.4
Modulus of elasticity of concrete (GPa)	23.1	39.1
Tensile strength of FRP rebar (MPa)	482.2	2130.0
Modulus of elasticity of FRP rebar (GPa)	26.2	178.0
FRP reinforcement ratio/ Balanced reinforcement ratio ($\rho_{f}/\rho_{fb}$)	0.263	12.700
Cracking moment/ Maximum service load moment ($M_{cr}/M_{a}$)	0.084	0.754
Moment of inertia of transformed cracked section/ Gross moment of inertia ($I_{cr}/I_{g}$)	0.032	0.314

**Table 3:** Typical material properties of GFRP and BFRP reinforcing rebars.

Type	Nominal Diameter (mm)	Average Tensile Strength (MPa)	Guaranteed Tensile Strength * (MPa)	Design Tensile Strength ** (MPa)	Design Tensile Strain (%)	Modulus of Elasticity (GPa)
GFRP	13.0	927.9	839.1	587.4	1.19	49.0
BFRP	13.0	1067.2	755.5	528.9	1.05	50.5

* Average tensile strength—3 × standard deviation [1] ** Environmental reduction factor ($C_{E})$ is applied with 0.7, exposed to earth and weather.

**Table 4:** Balanced reinforcement ratio of reinforced concrete slab.

Specimen	Balanced Reinforcement Ratio (%)	FRP Reinforcement Ratio/ Balanced Reinforcement Ratio
GFRP	0.626(0.421 *)	0.898(1.335 *)
BFRP	0.504(0.332 *)	1.115(1.693 *)

* Environmental reduction factor not applied in ACI 440.1R-15 [1].

**Table 5:** Result of experimental GFRP and BFRP rebar-reinforced concrete specimens.

Specimen	Initial Cracking Load (kN)	Ultimate Load (kN)	Spacing of Crack (mm)	Mode of Failure
GFRP-1	40.06	143.14	180–250	Shear–compressive
GFRP-2	37.28	132.86	150–240	Shear
BFRP-1	38.92	167.62	150–200	Compressive
BFRP-2	42.66	154.56	150–190	Shear–compressive

**Table 6:** Comparison of analytical deflections with experimental values at service load and ultimate load of GFRP specimens.

Reference	Service Load			Ultimate Load		
	GFRP1	GFPR2	Average	GFRP1	GFRP2	Average
Benmokrane et al. [28]	1.31	2.67	1.99	−10.46	−9.06	−9.76
Toutanji and Saafi [29]	5.94	6.65	6.29	−8.49	−7.92	−8.21
Hall and Ghali [30]	−3.30	−1.60	−2.45	−6.30	−6.05	−6.17
ISIS Canada Design manual [31]	−3.30	−1.60	−2.45	−6.30	−6.05	−6.17
ACI440.1R-03 [32]	5.96	6.43	6.19	7.18	8.19	7.69
Yost et al. [33]	3.34	4.34	3.84	−1.98	−1.14	−1.56
Bischoff [36,37]	2.47	4.61	3.54	−3.99	−3.56	−3.78
ACI 440.1R-06 [34]	4.11	4.97	4.54	−0.38	0.56	0.09
Rafi and Nadjai [35]	4.13	4.97	4.55	0.66	1.42	1.04
Bischoff and Gross [38]	2.47	4.61	3.54	−3.99	−3.56	−3.78
Mousavi and Esfahani [22]	1.59	2.86	2.23	−0.77	−0.36	−0.57
Neuyen et al. [39]	3.15	3.99	3.57	2.24	3.08	2.66
Proposed Model	0.78	2.02	1.40	0.26	0.51	0.38

**Table 7:** Comparison of analytical deflections with experimental values at service load and ultimate load of BFRP specimens.

Reference	Service Load			Ultimate Load		
	BFRP1	BFPR2	Average	BFRP1	BFRP2	Average
Benmokrane et al. [28]	−0.67	−1.52	−1.09	−9.22	16.36	3.57
Toutanji and Saafi [29]	4.81	3.75	4.28	−4.98	−13.30	−9.14
Hall and Ghali [30]	−5.05	−5.98	−5.52	−2.65	−10.99	−6.82
ISIS Canada Design manual [31]	−5.07	−6.00	−5.53	−2.72	−11.05	−6.88
ACI440.1R-03 [32]	6.25	4.44	5.34	8.30	1.01	4.65
Yost et al. [33]	1.83	0.65	1.24	−0.13	−7.98	−4.06
Bischoff [36,37]	−0.24	−0.88	−0.56	−0.74	−8.31	−4.82
ACI 440.1R-06 [34]	4.37	2.86	3.61	3.15	−4.38	−0.61
Rafi and Nadjai [35]	4.39	2.88	3.64	4.36	−3.36	0.50
Bischoff and Gross [38]	−0.24	−0.88	−0.56	−0.74	−8.91	−4.82
Mousavi and Esfahani [22]	0.26	−0.89	−0.32	2.83	−5.33	−1.25
Neuyen et al. [39]	2.69	1.20	1.94	3.85	−3.55	0.15
Proposed Model	−0.38	−1.61	−1.00	4.08	−4.22	−0.07

## Data Availability

Not applicable.

## Appendix A

**Table A1:** Database of the experimental four-point flexural test for deflection of FRP-reinforced concrete members.

No	Reference	Specimen	b	d	h	$f_{c}^{\prime }$	$f_{f}$	$E_{f}$	$A_{f}$	L	$L_{a}$
1	[21]	C212D1a	140.0	163.4	190.0	59.8	1353	63,232	226.5	1800	600
2		C212D1b	140.0	163.4	190.0	59.8	1353	63,232	226.5	1800	600
3		C216D1a	140.0	161.5	190.0	56.3	995	64,152	402.5	1800	600
4		C216D1b	140.0	161.5	190.0	56.3	995	64,152	402.5	1800	600
5		C316D1a	140.0	161.5	190.0	55.2	995	64,152	603.7	1800	600
6		C316D1b	140.0	161.5	190.0	55.2	995	64,152	603.7	1800	600
7		C212D2a	140.0	142.5	190.0	39.6	1353	63,252	197.5	1800	600
8		C212D2b	140.0	142.5	190.0	39.6	1353	63,252	197.5	1800	600
9		C216D2a	140.0	140.6	190.0	61.7	995	64,152	350.4	1800	600
10		C216D2b	140.0	140.6	190.0	61.7	995	64,152	350.4	1800	600
11		C316D2a	140.0	140.6	190.0	60.1	995	64,152	525.6	1800	600
12		C316D2b	140.0	140.6	190.0	60.1	995	64,152	525.6	1800	600
13	[40]	A1	150	165	200	46.2	1506	50,080	176.71	2610	870
14		B1	150	165.2	200	46.2	1506	50,080	265.07	2610	870
15		C1	150	153.5	200	46.2	1506	50,080	353.43	2610	870
16	[41]	B1-1	150	275	300	60	482.2	57,982	100.5	1600	300
17		B1-2	150	275	300	60	636.3	43,939	157.1	1600	300
18		B1-3	150	275	300	60	745.6	37,500	226.2	1600	300
19		B2-1	150	275	300	50	482.2	57,982	100.5	1600	300
20		B2-2	150	275	300	50	636.4	43,939	157.1	1600	300
21		B2-3	150	275	300	50	745.6	37,500	226.2	1600	300
22	[42]	FB-1	180	186.0	230	27	841	42,100	144	1600	685
23		FB-2	180	186.0	230	27	841	42,100	216	1600	685
24		FB-3	180	173.5	230	27	841	42,100	288	1600	685
25	[22]	B1	150	165	200	20	490	41,000	142	2000	650
26		B2	150	165	200	20	490	41,000	471	2000	650
27		B3	150	165	200	20	490	41,000	671	2000	650
28		B4	150	165	200	38	490	41,000	142	2000	650
29		B5	150	165	200	38	490	41,000	471	2000	650
30		B6	150	165	200	38	490	41,000	671	2000	650
31		B7	150	165	200	64	490	41,000	142	2000	650
32		B8	150	165	200	64	490	41,000	471	2000	650
33		B9	150	165	200	64	490	41,000	671	2000	650
34	[43]	BRC1	120	175.3	200	42.6	1676	135,900	147.2	1750	675
35		BRC2	120	175.3	200	41.7	1676	135,900	147.2	1750	675
36	[44]	GB1-1	180	268	300	35	695	40,000	250.8	2800	1200
37		GB1-2	180	268	300	35	695	40,000	250.8	2800	1200
38		GB2-1	180	268	300	35	695	40,000	381.1	2800	1200
39		GB2-2	180	268	300	35	695	40,000	381.1	2800	1200
40		GB3-1	180	225	300	35	695	40,000	445.5	2800	1200
41		GB3-2	180	225	300	35	695	40,000	445.5	2800	1200
42	[45]	2G12	230	254	300	40	1000	50,000	226.2	3700	1250
43		3G12	230	254	300	40	1000	50,000	339.3	3700	1250
44		3G16	230	252	300	40	1000	50,000	603.2	3700	1250
45	[46]	2	200	157.5	210	31.3	700	35,630	1134	2900	1250
46		3	200	210.7	260	31.3	886	43,370	507	2900	1250
47		4	200	247.5	300	40.7	700	35,630	567	2900	1250
48		5	200	197.5	250	40.7	700	35,630	1134	2900	1250
49	[47]	2D16-8S70-N	250	214	250	30	775	46,000	402	2000	800
50		2D16-10S110-N	250	212	250	30	775	46,000	402	2000	800
51		2D16-8S35-N	250	214	250	30	775	46,000	402	2000	800
52		2D16-10S55-N	250	212	250	30	775	46,000	402	2000	800
53		5D10-8S70-N	250	217	250	30	789	44,000	393	2000	800
54		5D10-10S110-N	250	215	250	30	789	44,000	393	2000	800
55		5D10-8S35-N	250	217	250	30	789	44,000	393	2000	800
56		5D10-10S55-N	250	215	250	30	789	44,000	393	2000	800
57		3D18-8S70-N	250	213	250	30	800	42,000	763	2000	800
58		3D18-10S110-N	250	211	250	30	800	42,000	763	2000	800
59		3D18-10S55-N	250	213	250	30	800	42,000	763	2000	800
60		3D18-10S55-N	250	211	250	30	800	42,000	763	2000	800
61		5D14-8S70-N	250	214	250	30	825	45,000	770	2000	800
62		5D14-10S110-N	250	212	250	30	825	45,000	770	2000	800
63		5D14-8S35-N	250	214	250	30	825	45,000	770	2000	800
64		5D14-10S55-N	250	212	250	30	825	45,000	770	2000	800
65	[48]	CC	230	184	250	75.9	2130	146,200	256	1900	800
66		GG	230	184	250	75.9	941	48,100	762	1900	800
67	[23]	BF1	127	286	305	38.6	724	26,200	253	3050	990
68		BF2	127	286	305	40.7	724	26,200	253	3050	990
69		BF3	127	286	305	40.0	724	26,200	379	3050	990
70		BF4	127	286	305	38.6	724	26,200	379	3050	990
71		BF5	127	286	305	37.2	724	26,200	506	3050	990
72		BF6	127	286	305	35.2	724	26,200	506	3050	990
73		BF7	127	273	305	32.4	724	26,200	632	3050	990
74		BF8	127	273	305	29.7	724	26,200	632	3050	990
75		BF9	127	273	305	29.7	724	26,200	758	3050	990
76		BF10	127	273	305	35.2	724	26,200	758	3050	990
77		BF11	127	273	305	39.3	724	26,200	885	3050	990
78		BF12	127	273	305	30.4	724	26,200	885	3050	990
79	[49]	A1	100	125	150	55.4	732	37,500	63.3	2400	900
80		A2	100	125	150	55.4	732	37,500	95.0	2400	900
81		A3	100	125	150	55.4	732	37,500	126.7	2400	900
82		B1	100	125	150	70.8	1764	55,600	142.7	2400	900
83		B2	100	125	150	70.8	1764	55,600	214.0	2400	900
84		B3	100	125	150	70.8	1764	55,600	285.3	2400	900
85		C1	100	125	150	90.1	1605	48,600	253.4	2400	900
86		C2	100	125	150	90.1	1605	48,600	380.0	2400	900
87		C3	100	125	150	90.1	1605	48,600	506.7	2400	900
88	[50]	BC2HA	130	160	180	57.2	773	38,000	241.28	1500	500
89		BC2VA	130	160	180	97.4	773	38,000	241.28	1500	500
90		BC4NB	130	140	180	46.2	773	38,000	504.14	1500	500
91		BC4HA	130	140	180	53.9	773	38,000	504.14	1500	500
92		BC4VA	130	140	180	93.5	773	38,000	504.14	1500	500
93	[51]	G-1	130	210	230	41.6	778	54,000	175	1900	1500
94		C-0-1	130	210	230	41.6	1875	178,000	175	1900	1500
95	[52]	40#2-0.5	100	127.8	150	40	732	37,500	63.4	2000	667
96		40#3-1.0	100	126.2	150	40	1764	55,600	142.6	2000	667
97		40#4-2.0	100	124.7	150	40	1605	48,600	253.4	2000	667
98		80#2-0.5	100	127.8	150	80	732	37,500	63.4	2000	667
99		80#3-1.0	100	126.2	150	80	1764	55,600	142.6	2000	667
100		80#4-2.0	100	124.7	150	80	1605	48,600	253.4	2000	667
101	[53]	B1-35-12	200	262	300	35	1166	65,000	226	2700	500
102		B2-35-16	200	262	300	35	1122	63,000	402	2700	500
103		B3-35-20	200	262	300	35	1117	69,000	628	2700	500
104		B4-35-25	200	262	300	35	1340	65,000	980	2700	500
105		B5-65-12	200	250	300	65	1166	65,000	226	2700	500
106		B6-65-16	200	250	300	65	1122	63,000	402	2700	500
107		B7-65-20	200	250	300	65	1117	69,000	628	2700	500
108		B8-65-25	200	250	300	65	1340	65,000	980	2700	500
109	Current study	GFRP-1	650	133.5	180	45.4	649.5	49,000	488	1800	600
110		GFRP-2	650	133.5	180	45.4	649.5	49,000	488	1800	600
111		BFRP-1	650	133.5	180	45.4	747.0	50,500	488	1800	600
112		BFRP-2	650	133.5	180	45.4	747.0	50,500	488	1800	600
